# Artificial intelligence in gifted and talented education: a scoping review

**DOI:** 10.3389/fpsyg.2026.1898467

**Published:** 2026-07-29

**Authors:** Omar Abdullah Alsamani, Yasir Ayed Alsamiri

**Affiliations:** 1 Department of Special Education, College of Education, University of Ha’il, Hail, Saudi Arabia; 2 Department of Education, Islamic University of Madinah, Madinah, Saudi Arabia

**Keywords:** artificial intelligence, ChatGPT, generative AI, gifted and talented education, machine learning, scoping review, talent development, twice-exceptional

## Abstract

**Introduction:**

Artificial intelligence (AI) is being increasingly adopted across gifted and talented education. However, the emerging literature remains dispersed across instructional, developmental, assessment, and ethical domains.

**Methods:**

This scoping review maps how AI has been studied in gifted and talented education, including the forms of AI examined, the populations and contexts represented, the educational purposes addressed, and the opportunities, risks, and gaps reported across the literature. Following a scoping review approach informed by PRISMA-ScR and the Population–Concept–Context framework, database searches and screening procedures identified 26 eligible studies.

**Results:**

The review identified two broad trajectories. The first is instructional and developmental, where generative AI, AI tutoring, and AI-generated materials are used or proposed to support differentiation, writing, questioning, creativity, mentoring, and personalized learning. The second is assessment-oriented, where machine learning and related models are applied to gifted and talented identification, twice-exceptional identification, and decision support. Across both trajectories, the literature positions AI less as a replacement for educators than as a tool whose value depends on task design, teacher judgment, and human oversight. Reported opportunities include support for differentiated preparation, advanced learning, creative production, and the recognition of complex learner profiles. The reported risks include bias in identification data, overreliance, threats to originality, privacy concerns, limited transparency, and insufficient preparation among educators and institutions.

**Discussion:**

Overall, the evidence base on AI in gifted and talented education remains at an early stage. The review suggests that AI holds potential to advance gifted and talented education. Future research should move toward longitudinal, comparative, and equity-centered studies, particularly in AI-supported identification and twice-exceptional education.

## Introduction

Artificial intelligence (AI) has entered education at a rapid pace, becoming part of educational practice and research. Machine learning and natural language processing had been earlier embedded in tutoring systems, learning analytics, and automated assessment, but the public release of large language models marked a turning point, placing generative tools directly in the hands of students and teachers with no technical training (Kasneci et al., 2023; Yan et al., 2024). Within a short period these tools were being used intensively for various tasks in education, including drafting materials, giving feedback, supporting writing, and tailoring content to individual learners, and educators began asking not whether AI would shape their work but how (Alsamani, 2026; Li et al., 2024).

These questions are especially pointed in gifted and talented education, a field built around aims that AI appears well positioned to serve. Gifted and talented learners typically need depth and complexity that move beyond grade-level expectations (Alsamani, 2023). Supporting these needs in regular classrooms requires differentiated instruction that extends beyond grade-level expectations and supports advanced learning (Siegle, 2023, 2024). Teachers of gifted and talented learners, however, report limited time and support for the individualized planning this requires (Alsamani, 2019; Ecker et al., 2025; Lewis, 2025). Generative AI is increasingly seen as a way to support what a classroom teacher can offer, drafting differentiated tasks, and interest-based materials that would otherwise take hours to prepare (Guilbault et al., 2025). These questions are also psychological in nature. They bear on gifted learners’ motivation, creativity, cognitive load, self-regulation, learner agency, anxiety, and socioemotional development, as well as on equity in educational decision-making—constructs that place the present review squarely within educational psychology.

Artificial intelligence also reaches a second and more consequential function: the identification of who is recognized as gifted and talented in the first place. Identification carries a long and unresolved equity problem, with Black, Latinx, English-learner, bilingual and second-language and low-income students consistently underidentified, owing in part to biased teacher referral and reliance on narrow test-based criteria (Card and Giuliano, 2016; Grissom and Redding, 2016). Machine learning has recently been advanced as a way to detect talent that conventional procedures miss, including in students whose profiles do not fit standard expectations (Hodges and Mohan, 2019; Lee et al., 2025). The hazard runs alongside the promise: algorithms learn from existing records and rating practices, so a model trained on historically skewed data can reproduce the disparities it is meant to reduce (Atmaca and Baloğlu, 2025; Lee et al., 2025). Instructional and assessment uses thus carry different stakes, since a weak AI-generated task can be revised by a teacher, whereas a flawed identification decision can determine access to services.

Twice-exceptional learners sit at the intersection of both concerns. Having advanced ability and disability at the same time can obscure one from the other. Thus, these students are easily missed by identification systems for gifted education and special education diagnoses and hard to serve through standard differentiation (Mahmoudi-Dehaki and Nasr-Esfahani, 2025; Ronksley-Pavia et al., 2025). AI has been explored both to scaffold their learning and to detect their uneven profiles, which makes them a revealing test of how far algorithmic support can responsibly extend.

Work across these areas has grown quickly but remains scattered. Studies fall into separate strands–generative AI for instruction, machine learning for identification, teacher readiness, twice-exceptionality–and they differ widely in purpose, design, and the type of AI examined, from controlled interventions to conceptual proposals. Existing studies and reviews tend to cover a single part, such as AI for gifted language learners (García-López et al., 2025), leaving the field without a synthesis that maps how AI is being studied across gifted and talented education. Without such a map, it is hard to see where AI is being accumulated and where it is missing.

The present study responds to this gap by mapping the literature on AI across gifted and talented education. It identifies the forms of AI that have been studied, the populations and contexts represented, the purposes AI has served, and the opportunities and risks reported across studies. In doing so, the review provides an organized account of an emerging field, showing where evidence is accumulating, where it remains limited, and under what conditions AI might be used responsibly, particularly in identification and for twice-exceptional learners. A scoping review is well suited to this purpose because scoping reviews are designed for emerging and heterogeneous fields where the goal is to clarify the scope and nature of the literature rather than estimate the effect of a single intervention (Arksey and O’Malley, 2005; Levac et al., 2010; Peters et al., 2020; Tricco et al., 2018). The review was guided by five questions:

RQ1. What types of AI, and in what applications, have been examined in gifted and talented education research?

RQ2. Which populations and contexts are represented in gifted and talented education research?

RQ3. For what purposes is AI positioned across the literature?

RQ4. What opportunities and risks are reported across the included studies?

RQ5. What gaps remain in the literature on AI and gifted and talented education?

## Materials and methods

### Design

This study utilized a scoping review design to investigate the emerging literature on artificial intelligence in gifted and talented education. A scoping review approach was selected because the field is at an early stage and includes heterogeneous forms of evidence. Scoping reviews are appropriate when the purpose is to clarify the scope, nature, and distribution of evidence in an emerging or conceptually diverse field rather than to estimate intervention effectiveness (Arksey and O’Malley, 2005; Levac et al., 2010; Peters et al., 2020).

The review was informed by established scoping review methodology and reported in alignment with the PRISMA-ScR framework (Tricco et al., 2018). The completed PRISMA-ScR checklist is provided in Supplementary Table 4. It was guided by the Population–Concept–Context (PCC) framework. This framework focuses on the development of eligibility criteria and the organization of the review question, consistent with JBI guidance for scoping reviews (Peters et al., 2020). The population, in this review, included gifted and talented learners, high-ability learners, twice-exceptional learners, academically advanced students, and educators working in gifted and talented education contexts. The concept was artificial intelligence, broadly defined to include generative AI, large language models, machine learning, AI tutoring, neural networks, classification algorithms, and AI-generated instructional materials. The context was gifted and talented education, including gifted classrooms, enrichment programs, gifted education institutes, talent development programs, twice-exceptional education, teacher preparation, and identification or assessment practices.

### Eligibility criteria

Studies were included if they met four criteria. First, the study had to address artificial intelligence as a central concept, tool, intervention, or analytic method. Second, the study had to be directly connected to gifted and talented education, gifted or talented learners, high-ability or academically advanced learners, twice-exceptional learners, gifted and talented education teachers, gifted and talented education programs, or identification practices relevant to gifted education. Third, the study had to be available in English. Fourth, the publication had to provide sufficient information for data charting, including the population or context, the type of AI examined, and the educational or assessment-related purpose.

As an operational decision rule, a study was included only when it explicitly involved gifted and talented education programs, formal gifted identification, high-ability programming, twice-exceptionality, gifted and talented education teachers, or a recognized gifted or talent-development framework. Studies addressing general high achievement, advanced academic performance, or AI in education broadly—without an explicit connection to gifted and talented education—were excluded, even when they used terms such as advanced learners or high-achieving students. The review included empirical studies, conceptual or theoretical papers, praxis-oriented articles, action research, methodological demonstrations, conference papers, and reviews when they directly addressed the intersection of AI and gifted and talented education. This inclusive approach was appropriate because scoping reviews commonly aim to map the breadth and nature of evidence in emerging or heterogeneous fields (Arksey and O’Malley, 2005; Peters et al., 2020).

Studies were excluded if AI was mentioned only incidentally or as part of a broader discussion of digital technology without a clear AI focus. Studies were also excluded if they used terms such as “talent,” “talented students,” “high-achieving students,” or “advanced learners” in a general academic performance sense without a clear connection to gifted and talented education or high-ability programming. Editorials, interviews, news items, association webpages, and non-scholarly professional materials were excluded from the final included studies, although some were used during screening to identify potentially relevant articles. Studies were also excluded if full eligibility could not be verified, if the publication was not in English, or if the text did not provide sufficient information for extraction.

### Information search strategy

A systematic search was conducted to identify literature examining the intersection of artificial intelligence and gifted and talented education. The search focused on multidisciplinary databases. The primary databases searched were Web of Science, Scopus, EBSCOhost, and ProQuest. The database search identified 397 records before duplicate removal: 104 from Web of Science, 113 from Scopus, 78 from EBSCOhost, and 102 from ProQuest (see Supplementary Table 1).

Additional targeted searches were conducted when reference lists or journal issue contents indicated potentially relevant articles that were not captured in the initial database search. Google Scholar was used as a supplementary verification source at the end of the search process. Approximately six targeted query combinations were run, pairing a gifted and talented term with an AI term (e.g., gifted AND “artificial intelligence”; gifted AND ChatGPT; “twice-exceptional” AND “machine learning”; “talent development” AND “generative AI”), and the first 50–100 results of each query were screened for relevance (approximately 400–500 records scanned in total). Backward and forward citation searching was also performed on the included studies. These supplementary methods were used to verify the completeness of the database search rather than as primary sources; they did not yield additional eligible studies beyond those already retrieved, and the 397 records therefore reflect the four databases only and were not augmented by Google Scholar or citation-search records.

The search combined AI-related terms with gifted and talented education terms. The AI-related terms were: “artificial intelligence” OR “generative AI” OR ChatGPT OR “machine learning” OR “large language model” OR “neural network” OR “AI tutoring.” These terms were combined with the following gifted education terms: gifted OR “gifted education” OR “talented education” OR “high ability” OR “high-ability” OR “twice exceptional” OR twice-exceptional OR 2e OR “talent development.” The core search strategy was adapted across databases while retaining conceptual consistency (Levac et al., 2010; Peters et al., 2020).

The search used broad terms because the literature on AI in gifted and talented education is emerging and uses inconsistent terminology. Some studies referred to gifted education, whereas others used terms such as high-ability learners, academically gifted students, talent development, or twice-exceptional learners. Similarly, AI was described using terms such as generative AI, ChatGPT, machine learning, and neural networks. Broad searching was therefore necessary to map the field as a scoping review.

Several adjacent technical and educational terms were also considered, including intelligent tutoring systems, adaptive learning, learning analytics, educational data mining, automated assessment, natural language processing, chatbot, deep learning, expert systems, and recommender systems; and, on the gifted education side, academically talented, high potential, advanced academic, giftedness, and talent identification. These adjacent terms informed the screening, interpretation, and supplementary verification of records, but they were not included as separate strings in the formal database search syntax. This is acknowledged as a search limitation, as relevant studies indexed exclusively under such terminology may have been missed.

The final search was completed in May 2026, and the search covered publications from January 2010 through May 2026. Although the search began in 2010 to capture earlier applications of machine learning and educational AI, most eligible studies were published after 2023, reflecting the rapid expansion of generative AI tools in education. The 2010 starting point was selected to capture earlier applications of machine learning and educational AI before the rapid expansion of generative AI after 2023.

### Screening and study selection

We exported and organized all retrieved records for screening. Duplicate records were removed before title and abstract screening. The initial database search identified 397 records across the searched databases (Web of Science, Scopus, EBSCOhost, and ProQuest; see Supplementary Table 1). After removing 155 duplicate records, 242 unique records remained for screening. Two reviewers independently screened titles and abstracts against the eligibility criteria, with particular attention to whether AI was a central focus and whether the study was directly connected to gifted and talented education, twice-exceptionality, high-ability learners, gifted and talented education teachers, or gifted and talented identification.

Initial agreement during title and abstract screening was 86%, with Cohen’s κ = 0.72. Disagreements at this stage mainly involved records that used broad terms such as talent, high-achieving students, advanced learners, or AI in a general educational context. Disagreements were resolved through discussion, and uncertain records were retained for closer review rather than excluded at the title and abstract stage. Because the search was intentionally broad, many records included general educational technology studies, AI studies in general education, studies of high-achieving students without a gifted and talented education focus, or machine learning studies focused on general academic prediction. These records were excluded when they did not meet the review’s Population–Concept–Context criteria, which were used to structure eligibility decisions in line with JBI scoping review guidance (Peters et al., 2020).

After title and abstract screening, 175 records were excluded and 67 were identified as direct candidates for closer review. The two reviewers independently examined these records using abstracts, full texts where available, and database metadata. Agreement increased during eligibility assessment to 92%, with Cohen’s κ = 0.84, reflecting the availability of more detailed information. Disagreements were resolved through discussion, by returning to the eligibility criteria, and by reviewing the full text. Following discussion and re-examination of all discrepant cases, both reviewers reached full agreement on the final set of 26 studies included in the review.

### Data charting and synthesis

A data-charting form was developed to collect key data from each included study for systematic charting of study characteristics (Levac et al., 2010; Peters et al., 2020). The extracted fields included author and year, country or context, publication type, evidence type, study design, population, sample characteristics, type of AI examined, educational or assessment purpose, key findings, reported opportunities, reported risks or challenges, reported limitations or interpretive cautions, and thematic relevance.

The two reviewers independently charted data from the 26 included studies. After independent charting, the extracted data were compared for consistency across key fields. Agreement across the main charted categorical fields ranged from 88% to 94%. Agreement was higher for descriptive fields, such as AI type, population, and publication type, and lower for more interpretive fields, such as thematic classification and evidence type. Discrepancies were resolved through discussion and by returning to the full text.

For empirical studies, charting focused on study design, participants, AI tool or method, educational context, outcomes, and findings. For conceptual, praxis-oriented, or methodological papers, charting focused on the proposed AI application, the gifted and talented education problem addressed, the implied educational purpose, and any cautions or implementation considerations. Review articles were charted as secondary evidence and were not treated as equivalent to primary empirical findings.

**FIGURE 1:**
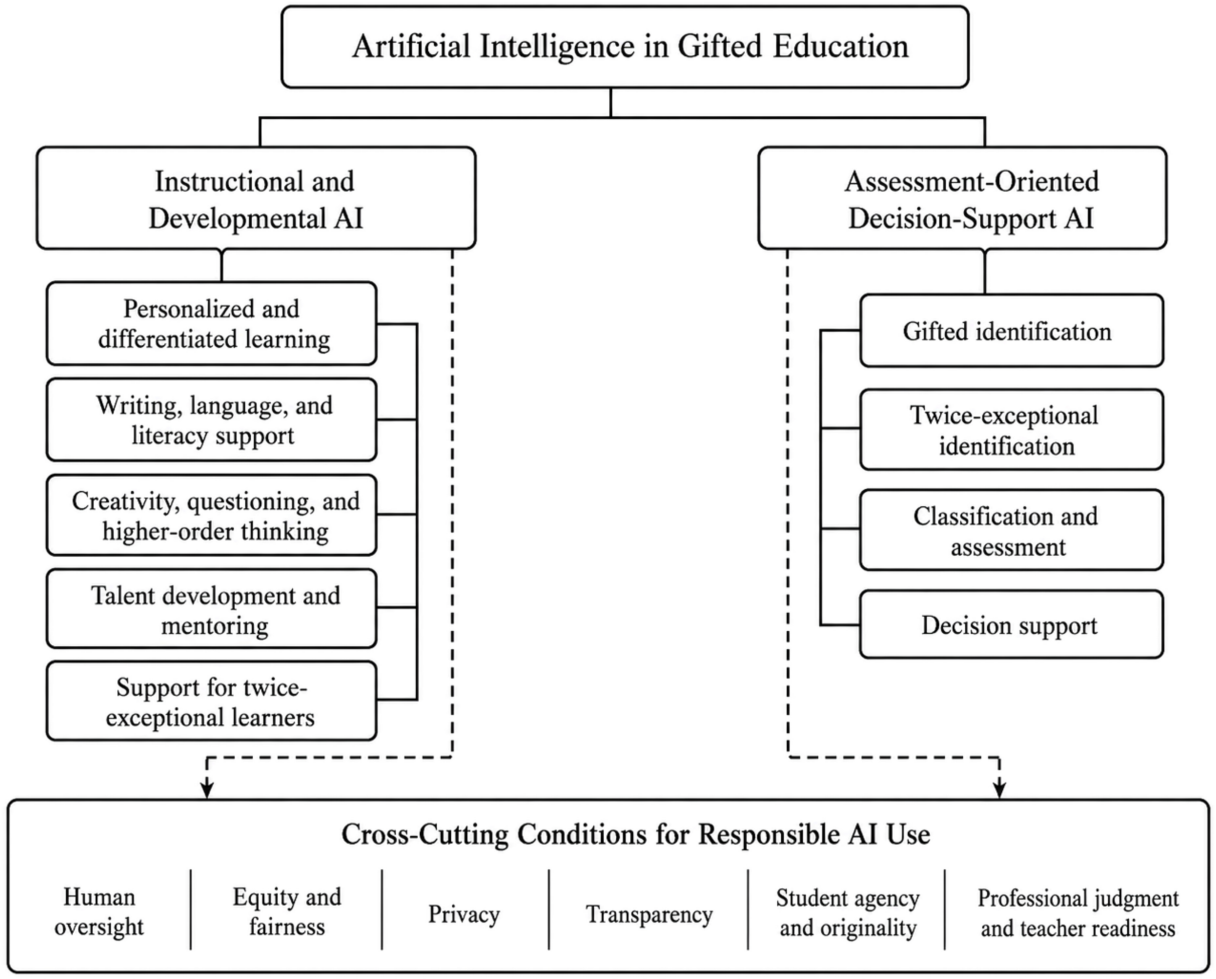
Emerging conceptual map of AI in gifted and talented education.

The synthesis of the literature followed a descriptive and thematic mapping approach, which is appropriate for scoping reviews (Arksey and O’Malley, 2005; Peters et al., 2020). First, studies were summarized descriptively by publication type, evidence type, population, AI type, and educational purpose. Second, patterns across the charted data were examined to identify recurring domains of AI use in gifted and talented education. This process resulted in six thematic categories: AI for personalized and differentiated learning; AI for writing, language, and literacy development; AI for creativity, questioning, and higher-order thinking; AI for identification, assessment, and decision support; AI for twice-exceptional and multi-exceptional learners; and teacher readiness, institutional practice, and ethical implementation. These themes were generated inductively from the charted data rather than imposed deductively from the research questions. Coding proceeded iteratively: the two reviewers independently grouped charted entries into candidate categories, compared their groupings, and refined them through discussion until reaching consensus, with disagreements resolved by returning to the full texts. The resulting themes were then organized into two higher-order trajectories and a set of cross-cutting conditions, as described in the Results and shown in Figure 1.

Studies were allowed to contribute to more than one theme when appropriate. For example, studies using ChatGPT to support writing were relevant to both language development and personalization, while machine learning studies focused on twice-exceptional identification contributed to both assessment and twice-exceptionality themes. The final thematic structure was used to organize the results and to identify gaps in the literature.

## Results

### Search results

**FIGURE 2:**
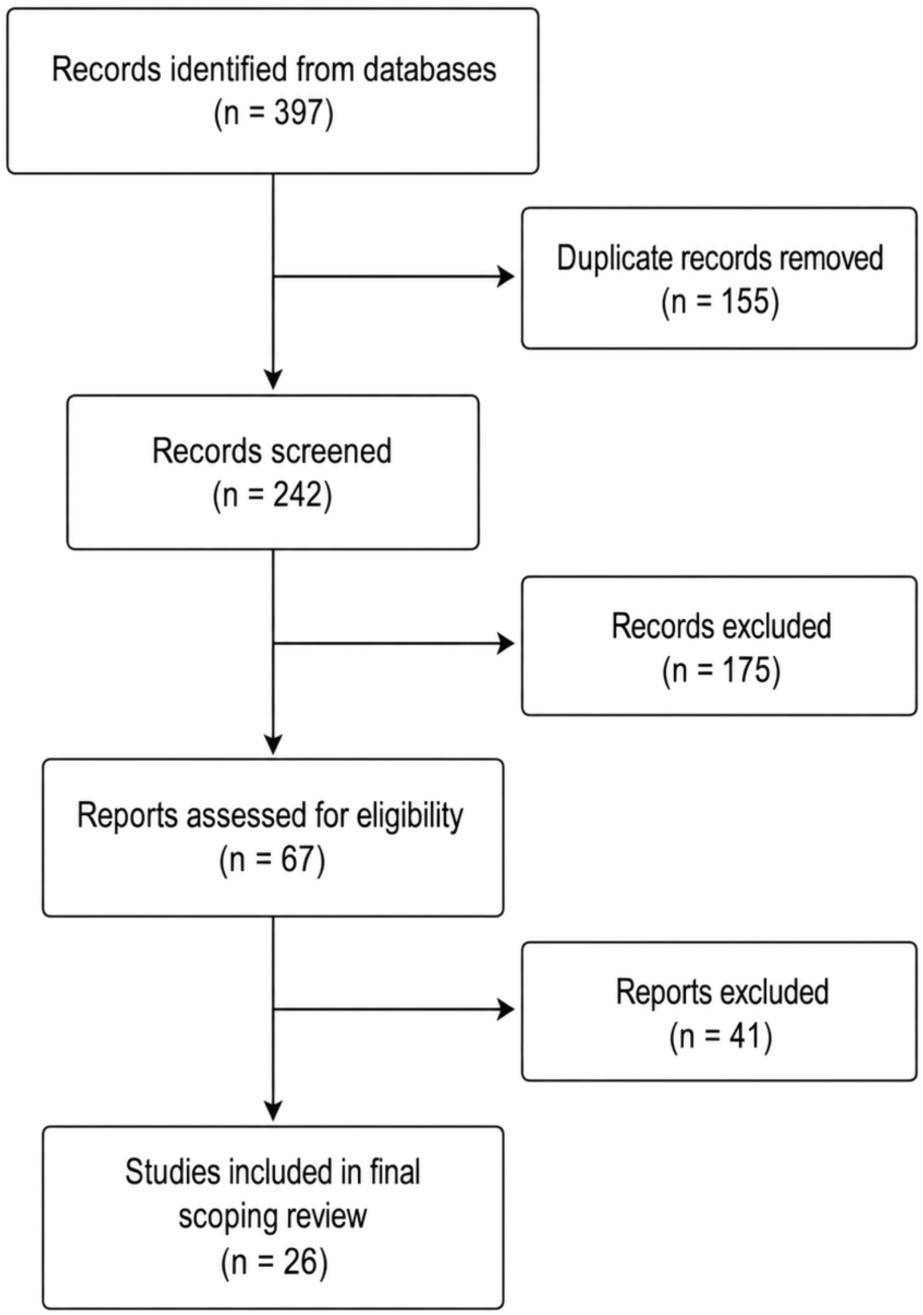
PRISMA flow diagram. Detailed reasons for the full-text exclusions (*n* = 41) are reported in Supplementary Table 2. The 397 records refer only to database records retrieved from Web of Science, Scopus, EBSCOhost, and ProQuest; Google Scholar and citation searching were used as supplementary verification procedures and were not added to this count.

Screening excluded records that addressed AI in general education without a gifted and talented education focus, studies of digital technology without a clear AI component, studies using talent or high achievement in a general academic sense, non-scholarly materials, editorials, and records outside the eligibility criteria. Sixty-seven records were retained as direct candidates for closer review. These records were examined through abstracts, full texts, and database metadata when available. Full-text eligibility assessment resulted in 41 studies being excluded, and 26 studies were selected as meeting the inclusion criteria for the final review (see Figure 2 and Supplementary Table 2).

The final set of included studies reflected the emerging and heterogeneous nature of the field. Included publications consisted of empirical studies, conceptual and praxis-oriented papers, action research, methodological demonstrations, conference papers, and one review. The diversity of publication types was retained because the purpose of the review was to map the field rather than synthesize effect sizes or evaluate a single intervention.

### Characteristics of included studies

**FIGURE 3:**
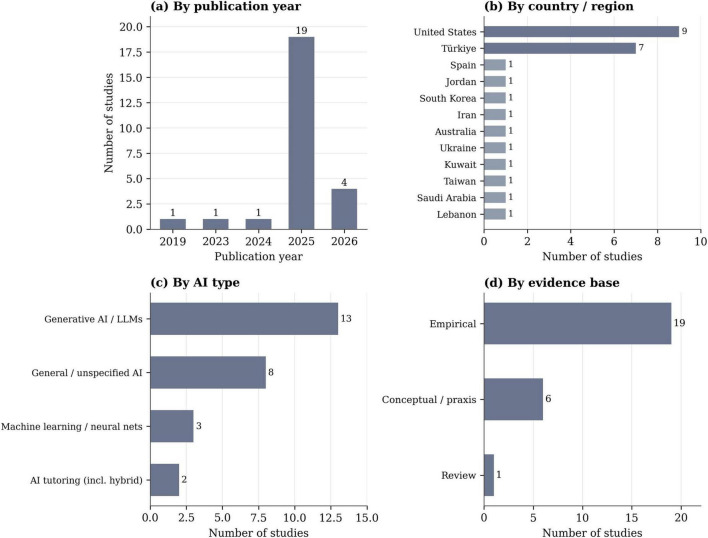
Characteristics of included studies by **(a)** publication year, **(b)** country or region, **(c)** AI type, and **(d)** evidence base.

The 26 included studies showed that research on AI in gifted and talented education is recent and rapidly expanding. The earliest included study focused on machine learning and neural networks in gifted identification, whereas most subsequent studies were published after the public emergence of widely accessible generative AI tools. The post-2023 literature increasingly addressed ChatGPT, generative AI, AI-generated instructional materials, AI tutoring, teacher readiness, and AI-supported identification. As shown in Figure 3a, publications were concentrated in 2025 (*n* = 19) and 2026 (*n* = 4), with single earlier studies appearing in 2019, 2023, and 2024.

The included studies varied: some were conceptual, theoretical, or praxis-oriented papers. Other studies were empirical studies examining AI-supported writing, STEM activities, creativity, student perceptions of AI, and AI use. A smaller group focused on assessment and identification, using machine learning, neural networks, classification algorithms, or teacher-rating data. Additional studies addressed twice-exceptional learners, gifted education teachers, and gifted education institutions. Table 1 summarizes the main characteristics of the included studies by author and year, design or publication type, population or context, AI type, main purpose, and thematic classification. By evidence base, the corpus comprised 19 empirical studies (drawing on collected qualitative, quantitative, or mixed data), 6 conceptual or praxis-oriented papers, and 1 systematic review (Figure 3d). By educational level, studies spanned primary and elementary settings (e.g., Doğan and Kahraman, 2025; Lee et al., 2025; Rubenstein et al., 2025), secondary classrooms (e.g., Guilbault et al., 2025), and college or university contexts (e.g., Hasanbaşoğlu and Baloglu, 2026; Mohamed et al., 2026), alongside studies of gifted educators and gifted education institutions (e.g., Lewis, 2025; Önal et al., 2026; Al-Bawaleez and Turkey, 2025). To preserve this distinction, the data-charting table classifies each study by evidence base and notes that conceptual and praxis-oriented papers indicate how the field frames AI rather than providing empirical demonstrations of effectiveness (see Supplementary Table 3). In terms of study design, the empirical studies spanned qualitative, mixed-methods, quasi-experimental, single-case, correlational, and machine-learning analyses, alongside conceptual, theoretical, praxis-oriented, methodological, and review papers. In terms of educational purpose, the studies addressed personalized and differentiated learning, writing and literacy support, creativity and higher-order questioning, gifted and twice-exceptional identification and decision support, and teacher readiness and institutional practice (see Table 1 and Supplementary Table 3).

**TABLE 1:** Characteristics of included studies.

ID	Author/year	Country	Design / type	Population / context	AI type	Main purpose	Theme
1	Siegle, 2023	United States	Praxis article	Gifted education	ChatGPT / AI	Differentiation support	T1
2	Siegle, 2024	United States	Praxis article	Talent development	AI tools	Acceleration, depth, interests	T1
3	Ecker et al., 2025	United States	Theoretical framework	Gifted programs	AI tutoring	AI tutoring model	T1
4	Lewis, 2025	United States	Praxis article	Gifted educators	GenAI	Teacher preparation	T6
5	Guilbault et al., 2025	United States	Classroom application	Secondary gifted classroom	ChatGPT	Personalized learning and mentoring	T1
6	Siegle, 2025	United States	Instructional strategy	Gifted students	Prompt engineering	Improve questioning	T3
7	Tamim, 2025	Lebanon	Mixed methods	Gifted and average-IQ students	AI / ChatGPT	Compare AI use	T1, T3
8	Bedir et al., 2025	Türkiye	Qualitative	Gifted students	AI	Explore perceptions	T3
9	Mohamed et al., 2026	Saudi Arabia	Quantitative correlational	Gifted EFL university students	GenAI	Motivation and anxiety	T1
10	Hasanbaşoğlu and Baloglu, 2026	Türkiye	Qualitative	Gifted college students	ChatGPT	Academic writing	T2
11	Li et al., 2025	Taiwan	Comparative quantitative study	Gifted and non-gifted students	AI	Scientific inquiry	T3
12	Alkan, 2026	Türkiye	Exploratory pre-post study	Gifted students	AI / ML	Cognitive load	T1, T3
13	Almumen and Jouhar, 2025	Kuwait	Single-case design	Gifted students	ChatGPT 3.5	Persuasive writing	T2
14	Doğan and Kahraman, 2025	Türkiye	Case study / pre-post + interviews	Gifted fourth graders	ChatGPT / AI-generated STEM	Scientific creativity	T3
15	Chernenko and Lysenko, 2025	Ukraine	Conference paper	Gifted youth	AI projects	Cognitive competence	T3
16	Ronksley-Pavia et al., 2025	Australia	Exploratory empirical study	2e / multi-exceptional learners	GenAI / LLMs	Personalized learning	T5, T1
17	Mahmoudi-Dehaki and Nasr-Esfahani, 2025	Iran	Mixed methods intervention	2e bilingual students	Human–AI tutoring	Writing support	T5, T2
18	Atmaca and Baloğlu, 2025	Türkiye	Assessment development	TD, gifted, 2e, SLD students	CART / ML	2e identification	T4, T5
19	Hodges and Mohan, 2019	United States	Methodological demonstration	Gifted identification	Neural networks	Classification demonstration	T4
20	Lee et al., 2025	South Korea	ML analysis	Elementary students (South Korean sample)	GMM / SVM / MLP	Improve identification	T4
21	Önal et al., 2026	Türkiye	Mixed methods	BILSEM teachers	AI	AI self-efficacy	T6
22	Al-Bawaleez and Turkey, 2025	Jordan	Qualitative	Gifted education institutes	AI	Institutional practice	T6
23	Akdeniz et al., 2025	Türkiye/USA	Empirical evaluation	Gifted education context	ChatGPT / Bard–Gemini	Generate Bloom questions	T3, T6
24	García-López et al., 2025	Spain	Systematic review	Gifted EFL / EL classrooms	AI	Review language learning uses	T2
25	Rubenstein et al., 2025	United States	Action research	Grades 3–4 / Project Brilliance	ChatGPT	Personalized playscripts	T2, T1
26	Thompson et al., 2025	United States	Quasi-experimental	Advanced learning virtual classroom	GPT-4o / LLM	Co-tutoring impact	T1

Theme codes: T1 = Personalized and differentiated learning; T2 = Writing, language, and literacy development; T3 = Creativity, questioning, and higher-order thinking; T4 = Identification, assessment, and decision support; T5 = Twice-exceptional and multi-exceptional learners; T6 = Teacher readiness, institutional practice, and ethical implementation. Studies could contribute to more than one theme, so multiple codes indicate cross-theme relevance. Country reflects the location of the study sample for empirical work and the first author’s affiliation for conceptual or review papers; Akdeniz et al. (2025) reflects a Türkiye–United States collaboration, Mohamed et al. (2026) a multinational Gulf collaboration (Saudi Arabia and Bahrain), and Lee et al. (2025) analyzed a South Korean sample from a United States institution.

The included studies were geographically concentrated. Of the 26 studies, 9 (35%) originated from the United States and 7 (27%) from Türkiye, together accounting for roughly three in five studies. The remaining studies were distributed across ten further contexts, with one study each from Lebanon, Saudi Arabia, Taiwan, Kuwait, Ukraine, Australia, Iran, South Korea, Jordan, and Spain (see Figure 3b). Two studies reflected cross-national collaboration (Akdeniz et al., 2025, Türkiye–United States; Mohamed et al., 2026, a Gulf collaboration spanning Saudi Arabia and Bahrain), and Lee et al. (2025) analyzed a South Korean student sample from a United States institution. No included studies originated from Sub-Saharan Africa, Latin America, or South Asia. This concentration in a small number of national contexts—particularly the United States and Türkiye—should be considered when interpreting the transferability of findings, because identification practices, gifted and talented education provision, and access to AI tools vary substantially across educational systems.

Generative AI tools were the dominant AI technologies in the recent literature. Machine learning, neural networks, classification and regression trees, and other algorithmic approaches appeared primarily in studies focused on identification, assessment, or classification. AI tutoring and AI-generated instructional materials appeared in studies focused on personalization, twice-exceptional learners, and differentiated learning experiences. Across the included studies, AI was used or proposed for several educational purposes: personalized learning, writing support, question generation, mentoring, STEM activity design, scientific creativity, cognitive competence, gifted identification, twice-exceptional identification, teacher preparation, and institutional transformation. Figure 3c summarizes the distribution of AI types across the included studies.

### Thematic findings

The data analysis led to six overlapping themes, revealing two overarching trajectories: instructional and developmental applications (e.g., personalization, literacy, creativity), and assessment-oriented decision support (e.g., gifted and twice-exceptional identification). Figure 1 provides an emerging conceptual map of these two trajectories and the cross-cutting conditions that shape responsible AI use across both.

The conceptual map in Figure 1 was derived inductively from the thematic charting rather than specified *a priori*. After the six themes were established, the two reviewers grouped them into two higher-order trajectories—instructional and developmental applications, and assessment-oriented decision support–on the basis of the educational purpose each theme served. The cross-cutting conditions shown at the base of the map (human oversight; equity and fairness; privacy; transparency; student agency and originality; and professional judgment and teacher readiness) were not a separate theme but recurring considerations that appeared across the charted opportunities and risks, most explicitly within the teacher-readiness and ethical-implementation theme. The map was refined iteratively as charting proceeded: trajectories and conditions were revised when additional studies were charted, until the structure accounted for all 26 studies without forcing any study into a single exclusive category. The study-level thematic matrix and evidence profiles are provided in Supplementary Table 5. The map therefore represents an analytic summary of the charted data rather than a pre-existing framework. The two trajectories also differ in their evidence basis: the instructional and developmental trajectory rests largely on conceptual, praxis-oriented, and small-scale empirical work, whereas the assessment-oriented trajectory rests on a small number of empirical machine-learning studies. Readers should therefore interpret the map as an organizing framework rather than as a claim of equivalent empirical support across all branches.

**FIGURE 4:**
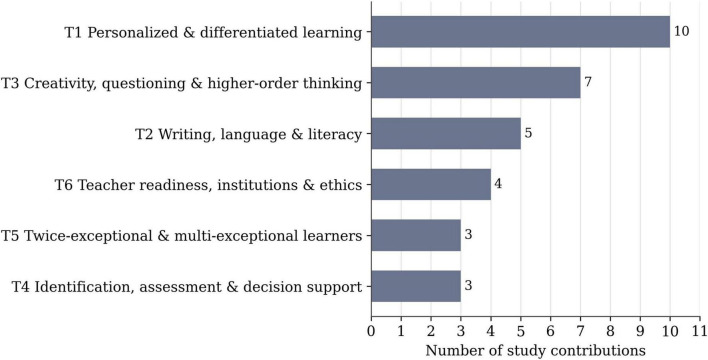
Distribution of included studies across the six themes.

Figure 4 shows how the included studies were distributed across the six themes. Because studies could contribute to more than one theme, the total number of thematic contributions (32) exceeds the number of studies (26).

Theme 1: AI for Personalized and Differentiated Learning

Alkan (2026) examined a gamified AI-based learning environment for gifted students, with cognitive load and achievement as the main outcomes. Personalized and differentiated learning was one of the most visible domains in the included literature. AI was introduced to support teachers’ abilities in providing advanced and interest-based learning opportunities for gifted students (Ecker et al., 2025; Guilbault et al., 2025; Siegle, 2023, 2024; Thompson et al., 2025). Across this theme, AI was framed as a tool that may extend teachers’ instructional capacity, not as a substitute for professional judgment.

This theme appeared in both conceptual and practice-oriented work. Siegle (2023, 2024) emphasized the role of AI in supporting the learning of gifted and talented learners in many aspects (e.g., advanced content, academic acceleration, depth and complexity, and interest-based learning). Guilbault et al. (2025) discussed how ChatGPT could support personalized learning of gifted learners and virtual mentoring in secondary gifted and talented classrooms. Ecker et al. (2025) proposed a four-dimensional AI tutoring framework suggesting that AI may support differentiation for both cognitive and socioemotional dimensions of gifted and talented learning. Thompson et al. (2025), however, offered a more cautious empirical contribution. Their quasi-experimental study of an LLM co-tutor in an advanced virtual science classroom found no significant differences in learning outcomes between students who used the LLM and those who did not, while showing that students’ interactions with AI required clearer structure and guidance.

Theme 2: AI for Writing, Language, and Literacy Development

Writing, language, and literacy formed a distinct area of AI application in the included studies. Studies examined Generative AI such as ChatGPT as a brainstorming tool, a writing support, a source of individualized feedback, and a teacher-facing resource for generating literacy materials aligned with students’ interests and reading levels (Almumen and Jouhar, 2025; García-López et al., 2025; Hasanbaşoğlu and Baloglu (2026); Rubenstein et al., 2025).

The writing-focused studies showed different forms of AI use. Almumen and Jouhar (2025) examined ChatGPT as a prewriting brainstorming tool for gifted and talented students’ persuasive writing and reported improvement across participants, while maintaining the view that AI should complement rather than replace students’ own writing processes. Hasanbaşoğlu and Baloglu (2026) focus on gifted college students’ use of ChatGPT in academic writing. Their findings showed that gifted students used AI for project planning, initial drafting, and revision, gaining efficiency, adaptability, and incidental learning. At the same time, the study highlighted concerns about related ethical issues such as the integrity, reliability, originality, and dependence on AI among gifted learners.

The literacy-focused work by Rubenstein et al. (2025) offered a different model of use. Instead of positioning students as direct users of AI, teachers used ChatGPT to generate personalized playscripts based on students’ interests and their reading level. This shifts AI from a student writing assistant to a curriculum-design tool. In this case, AI supported literacy engagement by helping students see their own imagined worlds transformed into texts.

Theme 3: AI for Creativity, Questioning, and Higher-Order Thinking

This theme is central to gifted and talented education since it moves beyond access to advanced content and focuses on how students think and produce ideas. Within the included studies, AI was examined as a tool for different tasks such as generating challenging questions, supporting inquiry-based science learning, designing STEM activities, developing project-based AI work, and strengthening students’ ability to interact critically with AI-generated responses (Akdeniz et al., 2025; Bedir et al., 2025; Chernenko and Lysenko, 2025; Doğan and Kahraman, 2025; Li et al., 2025; Siegle, 2025).

Several studies considered AI as a supportive tool for thinking rather than a source of completed answers. Akdeniz et al. (2025) examined whether AI tools could generate questions aligned with Bloom’s Taxonomy and found that AI-generated questions were stronger at lower cognitive levels, whereas human-written questions were stronger at the higher levels of Bloom’s Taxonomy. This finding is important for gifted and talented education because it suggests that AI can reduce some of the workload involved in question generation, although it still requires expert review when the goal is complex. Siegle (2025) approached the same issue from the learner side, arguing that gifted students’ questioning can be strengthened through prompt engineering, prompt chaining, and more deliberate follow-up questions.

Empirical studies in STEM and inquiry contexts added another layer to this theme. Doğan and Kahraman (2025) reported that AI-generated STEM activities improved scientific creativity among gifted fourth-grade students and were perceived positively by students. Chernenko and Lysenko (2025) examined gifted students’ participation in AI project development and connected this work with cognitive competence, including critical thinking, creative thinking, analytical skills, and scientific reasoning. Li et al. (2025) found that gifted and non-gifted students differed in their use of AI for scientific inquiry, with gifted students showing higher AI literacy and greater confidence in using AI safely.

These studies suggest that AI may support higher-order thinking when it is embedded in tasks that require students to question and extend ideas. Its value, though, is weaker when students treat AI output as an endpoint. For gifted and talented learners, the strongest use of AI appears to be as a thinking partner that provokes inquiry and refinement. These findings should be read cautiously because the tools and evidence base are still developing.

Theme 4: AI for Identification, Assessment, and Decision Support

Part of the literature investigated AI as a tool for identification, assessment, and decision support. Machine learning studies treated gifted identification as a classification problem and explored whether algorithms could detect patterns in student data that may be difficult to identify through traditional procedures (Atmaca and Baloğlu, 2025; Hodges and Mohan, 2019; Lee et al., 2025).

Hodges and Mohan (2019) introduced machine learning to gifted education through a neural network demonstration, framing classification as a possible area for future research while also cautioning against direct and uncritical application in gifted identification. More recent work extended this direction empirically. Lee et al. (2025) examined three models (Gaussian Mixture Models, support vector machines, and multilayer perceptron models) with teacher rating scale data of elementary students. The results indicated that machine learning could potentially support clustering and classification of gifted individuals. However, implementing these models within gifted identification revealed challenges related to underrepresented students and teacher rating bias across socioeconomic and ethnic groups.

The identification-focused literature suggests that AI may help broaden the evidence used in gifted education decisions, particularly when student profiles are complex or uneven. This is especially relevant to twice-exceptional identification, where strengths and disabilities may mask one another. At the same time, assessment-oriented AI carries higher stakes than instructional AI. Errors in a generated learning activity can be revised by a teacher; errors in identification may affect access to services. For this reason, AI in assessment should be understood as decision support, not decision replacement.

Theme 5: AI for Twice-xceptional and Multi-Exceptional Learners

Several included studies focused on twice-exceptional and multi-exceptional learners. Although this was a smaller area of the literature, it is particularly important because these learners often present uneven profiles showing advanced strengths along with learning challenges or neurodivergent characteristics.

A study by Ronksley-Pavia et al. (2025) explored the use of GenAI to create personalized learning approaches for twice-exceptional (2e) and multi-exceptional neurodivergent students. Their work was used to prepare pedagogy through synthetic learner profiles before classroom implementation. The results demonstrated GenAI as a tool for helping educators think through complex 2e learner needs. Mahmoudi-Dehaki and Nasr-Esfahani (2025) provided an intervention example through human–AI hybrid tutoring for 2e bilingual students with motor dysgraphia and ADHD. The study results found improvement in writing-related outcomes, including handwriting fluency, orthographic coding, fine motor control, and composition skills. Students also held positive attitudes regarding the hybrid tutoring as it offered adaptive support and socio-emotional assistance. The overall results of this study suggest that AI is more useful when it is embedded within human-guided support.

The 2e identification literature adds another dimension. Because twice-exceptional learners may be overlooked when disability-related difficulties mask gifted potential, AI-supported classification may help detect complex patterns in student profiles. However, this same complexity also makes 2e learners vulnerable to misclassification if AI systems rely on incomplete data or rigid categories. Across this theme, the most defensible role for AI is therefore not to simplify 2e learners into fixed labels, but to support more responsive interpretation of their strengths, needs, and learning conditions.

Theme 6: Teacher Readiness, Institutional Practice, and Ethical mplementation

The final theme focused on the conditions that shape meaningful utilization of AI in gifted education. Within the investigated literature, successful AI integration depended on teachers’ readiness, institutional support, ethical awareness, and the ability to connect AI use with gifted education goals (Al-Bawaleez and Turkey, 2025; Lewis, 2025; Önal et al., 2026).

Studies in this theme held, in general, positive views of AI involvement, but they also pointed to practical and ethical constraints. Al-Bawaleez and Turkey (2025) found that teachers in gifted education viewed AI as useful for administrative efficiency. Önal et al. (2026) reported that teachers in gifted and talented education institutions had relatively high AI self-efficacy, especially in assistance and technological skills. At the same time, those teachers expressed caution about the ethical issues and the need for targeted professional development in this area. Lewis (2025) argued that gifted and talented educators need more than general awareness of GenAI; they need preparation that connects AI literacy with gifted and talented pedagogy, ethical practice, and professional judgment. This is especially important because AI can generate materials, modify lessons, and support differentiation, but it cannot determine whether those outputs are appropriate for a particular gifted learner without human interpretation.

Within this theme, responsible implementation emerged as a condition for all other uses of AI. Personalized learning, writing support, creativity, and identification all depend on teachers’ ability to evaluate AI outputs, protect student data, guide ethical use, and preserve student agency. In gifted and talented education, AI integration is therefore not mainly a technical issue. It is a professional and pedagogical issue shaped by teacher expertise, institutional policy, and human oversight.

### Findings by research question

The five research questions are answered directly in Table 2, which maps each question to the corresponding findings across the 26 included studies.

**TABLE 2:** Summary of findings by research question.

RQ	Research question	Summary of findings across the 26 studies
RQ1	Types of AI and applications	Generative AI / large language models dominate (13 studies), applied to differentiation, writing, questioning, mentoring, and STEM design; machine learning and neural networks (3) are used for identification and classification; AI tutoring and human–AI hybrid tutoring (2) for personalization and 2e support; general or unspecified AI (8) across perceptions, motivation, and review work.
RQ2	Populations and contexts	Gifted, high-ability, and academically advanced learners from primary through university level; twice-exceptional learners; gifted educators; and gifted education institutions. Contexts include regular and enrichment classrooms, science-and-art centers (BILSEM), virtual classrooms, and identification settings. Studies are geographically concentrated in the United States (9) and Türkiye (7).
RQ3	Purposes AI is positioned to serve	Personalized and differentiated learning; writing and literacy support; creativity and higher-order questioning; mentoring; STEM activity design; gifted and twice-exceptional identification and decision support; and teacher preparation and institutional practice.
RQ4	Reported opportunities and risks	Opportunities: differentiated and faster preparation, advanced and creative production, recognition of complex learner profiles, and efficiency gains. Risks: bias in identification data, overreliance, threats to originality and academic integrity, privacy concerns, limited transparency, and insufficient teacher and institutional readiness.
RQ5	Remaining gaps	Few longitudinal, experimental, or comparative studies; limited equity-focused validation of AI-supported identification; little dedicated twice-exceptional research; a narrow geographic and English-language evidence base; and a predominance of conceptual over robust empirical work.

## Discussion

This review mapped 26 studies on AI in gifted and talented education, and the clearest pattern is that AI is not entering the field along a single path. Two trajectories run through the literature: one instructional and developmental, the other assessment-oriented. Holding them apart matters, because each carries different evidence requirements and a different ethical weight. Framed within educational psychology, both trajectories bear directly on core constructs—motivation, creativity, cognitive load, self-regulation, learner agency, anxiety, and equity in educational decision-making—that shape how gifted and talented learners experience AI-supported instruction and assessment.

In the first trajectory, AI supports teaching and learning. The studies here treat it as a way to differentiate instruction, support writing, extend mentoring, and prompt advanced questioning and creative work (Ecker et al., 2025; Guilbault et al., 2025; Rubenstein et al., 2025; Siegle, 2023, 2024; Thompson et al., 2025). These uses line up with familiar aims in gifted and talented education–depth, acceleration, autonomy, interest-based learning–and their appeal is practical, since they can ease the preparation that differentiation usually demands.

The second trajectory is narrower but more consequential. Here AI informs classification and identification, with machine learning applied to rating-scale data, cognitive batteries, and related profiles (Atmaca and Baloğlu, 2025; Hodges and Mohan, 2019; Lee et al., 2025). The stakes differ in kind, not only degree: an error in a generated activity can be corrected by a teacher, while an error in identification can shape whether a child reaches gifted and talented services at all. For that reason the most defensible role across both trajectories is supervised augmentation –AI-generated differentiation is not automatically good differentiation, and algorithmic identification is not automatically equitable identification.

We use the term supervised augmentation to denote a specific division of labor in which AI extends rather than replaces professional capacity, and in which a qualified educator or evaluator retains authority over consequential decisions. The concept draws on two established ideas in human–AI interaction. The first is the distinction between augmentation and automation: augmentation positions AI as a tool that enlarges what a professional can do, rather than as an autonomous agent that acts in their place (Amershi et al., 2019). The second is the human-in-the-loop principle that AI outputs should be treated as imperfect and contestable, with interfaces and workflows designed so that experts can inspect, question, and override them (Cai et al., 2019). Supervised augmentation is narrower than the broad notion of human-in-the-loop AI: it specifies not only that a human is present in the workflow, but that the human holds final responsibility for pedagogical and identification judgments, that AI outputs enter the process as advisory evidence rather than as determinations, and that the professional’s role is to evaluate, contextualize, and where necessary reject those outputs. In gifted and talented education this means that AI-generated differentiation is reviewed for appropriateness and challenge before use, and that algorithmic identification functions as one input among several subject to human review rather than as a screening decision in its own right.

### AI, creativity, and student agency

The creativity studies point to a conditional benefit. AI-generated STEM tasks, Bloom-aligned questions, and prompt-based work were reported as ways to stimulate idea generation and inquiry (Akdeniz et al., 2025; Doğan and Kahraman, 2025; Siegle, 2025), but the value seems to depend on how the task is built rather than on the tool. When students use AI to generate alternatives, constraints, or counter-questions and then critique and extend them, the intellectual work stays with the learner; when AI supplies finished answers, that work may be reduced (Chernenko and Lysenko, 2025; Li et al., 2025). This is why student agency is the central design question for instructional AI: gifted and talented learners need to remain the authors and evaluators of their own work, even as AI lowers the effort of producing a first draft.

### AI-supported identification of gifted and twice-exceptional learners

Identification is where promise and risk are sharpest, because it is tied to access. Machine learning may surface patterns in cognitive or rating-scale data that traditional procedures miss, which is especially relevant for learners whose profiles fall outside conventional expectations–twice-exceptional students, multilingual learners, and those from underrepresented groups (Atmaca and Baloğlu, 2025; Lee et al., 2025). But these models learn from existing records and rating practices, and Lee et al. (2025) found exactly the bias such records can carry across socioeconomic and ethnic lines. The promise lies in making patterns more visible; the danger lies in treating them as neutral or final. On current evidence, AI in identification is best understood as advisory, one input among several alongside human review. A more rigorous treatment requires attention to several specific risks. Algorithmic fairness cannot be assumed: models trained on historical referral and rating data can encode the demographic disparities documented in gifted identification (Card and Giuliano, 2016; Grissom and Redding, 2016), and Lee et al. (2025) observed rating bias across socioeconomic and ethnic groups in precisely the kind of data such models use. Model transparency matters because opaque classifiers make it difficult for educators to understand, question, or contest a recommendation. Validity must be examined across demographic subgroups rather than only in aggregate, and the representativeness of training data should be reported, since underrepresented groups are most likely to be misclassified. Concretely, programs that pilot algorithmic screening should validate models on local and demographically diverse samples, report subgroup performance, document the data and features used, retain human review over every consequential decision, and monitor for disparate impact over time. Without these safeguards, AI-supported identification risks reproducing the very inequities the field seeks to reduce.

### Twice-exceptional learners as a sharp case

Twice-exceptional learners concentrate both sides of this picture. Their uneven profiles are what AI scaffolding might serve well–Mahmoudi-Dehaki and Nasr-Esfahani (2025) reported gains from hybrid human–AI tutoring, and Ronksley-Pavia et al. (2025) used synthetic profiles to help teachers rehearse support. Yet the same unevenness is what a model can flatten, misreading masked ability or compressing a complex profile into a fixed label. The knowledge held by families, specialists, and the students themselves is not something a model output captures, which is why this group marks the limit of what algorithmic interpretation should decide on its own. For twice-exceptional learners in particular, AI may support interpretation and individualized planning, but it should not replace multidisciplinary assessment, teacher expertise, family knowledge, specialist input, or the student’s own perspective. Because their profiles are uneven and easily distorted by incomplete or biased data, any algorithmic output should be treated as one provisional source of evidence within a broader, human-led process.

### Teacher readiness and professional judgment

A consistent thread across themes is that teacher expertise becomes more important as AI enters the work, not less. AI can shorten the time needed to draft instructional options, but the judgment of whether an option is accurate, sufficiently challenging, and appropriate for a particular gifted and talented learner cannot be delegated to it (Al-Bawaleez and Turkey, 2025; Lewis, 2025; Önal et al., 2026). In that sense AI sharpens rather than reduces the specialized role of the gifted educator, who now has to combine knowledge of these learners with critical evaluation of what the tools produce.

### Ethical and equity considerations

Equity and ethics cut across every theme. A recurring concern is that machine learning is read as more objective than it is, when its outputs reflect the data, labels, and design choices behind it–a problem that matters most in identification, where it can reproduce the very inequities the field is trying to reduce (Atmaca and Baloğlu, 2025; Lee et al., 2025). Comparable concerns about transparency, bias, and privacy run through broader reviews of AI in education (Li et al., 2025; Yan et al., 2024). For a field whose aims include independence and originality, the ethical task is to keep AI use transparent and to protect the intellectual effort it can so easily replace. A more specific ethical agenda is required for gifted and talented contexts. On the instructional side, this includes child data privacy and meaningful consent, data governance over what student information is shared with commercial models, the explainability of AI outputs, and the risk of hallucinated or fabricated content presented as authoritative. On the assessment side, where decisions affect access to services, safeguards should include mandatory human review of consequential decisions, demographic auditing and bias testing, validation of model performance across subgroups, transparency for families and students about how AI informed a decision, and clear appeal mechanisms. Treating model outputs as advisory evidence rather than determinations is the minimum condition for using AI responsibly with this population.

### Implications for gifted and talented education practice

The evidence reviewed here is early and uneven, so its practical value lies less in prescribing particular tools than in clarifying how gifted educators might adopt AI without compromising the goals of the field. Several directions follow from the included studies. In everyday instruction, AI is most useful for rapid preparation rather than delivering finished teaching. The more durable gain comes from task design. Activities that ask students to critique, revise, or extend what AI produces keep the intellectual work with the learner; activities that treat AI output as a finished answer tend to erode it.

Where AI informs identification, it belongs in the process as one source of evidence among several rather than as the decision itself. Programs that experiment with algorithmic screening should be explicit about what data the model uses, monitor how it performs across demographic groups, and keep human review over every consequential decision, especially for students already underrepresented in gifted services.

Twice-exceptional learners call for the most caution. AI scaffolds for writing, organization, or pedagogical rehearsal can help teachers respond to uneven profiles, yet a model that compresses those profiles into fixed categories may obscure the interaction between strength and difficulty that defines this group. Decisions about 2e learners are sounder when AI sits alongside the knowledge of specialists, families, and the students themselves. These uses depend on preparation that current programs rarely provide. At the institutional level, principle-based policies covering disclosure, acceptable use, data privacy, and teacher review of AI-generated materials remain useful as tools change, whereas platform-specific rules date quickly.

### Future research directions

The studies reviewed here establish that AI is being used and proposed across gifted and talented education, but they say little about whether it works, for whom, and under what conditions. Most studies are conceptual and exploratory, so the clearest priority is a shift from demonstrating feasibility toward testing the conditions under which AI supports gifted and talented learners. Experimental and longitudinal designs are central to this shift: short studies can show engagement or immediate gains, but only sustained ones can show whether AI-supported learning produces durable development. Comparative work would also help clarify whether AI-based differentiation offers anything beyond established approaches, and how it changes the learning process rather than only test scores.

Twice-exceptional learners deserve dedicated attention. Because their profiles are uneven, group averages can obscure the variability that matters most, and designs such as single-case studies, mixed methods, and well-documented interventions are better suited to capturing it. Research here might examine AI support for writing, executive functioning, organization, and identification, while attending to how strength and difficulty interact in a given learner.

Identification research carries the highest stakes and needs the most scrutiny. Accuracy is not sufficient on its own. Studies should report how algorithmic tools perform across demographic groups, how educators interpret their outputs, whether such systems narrow or widen existing inequities, and how families and students experience decisions informed by them.

### Limitations

Several limitations should be taken into consideration. The search was deliberately broad, but the field is moving quickly, and relevant studies may have appeared shortly after the search date given the pace of generative AI in education. The review was also restricted to English-language publications, which may have excluded work from contexts where gifted and talented education and AI are developing actively.

The included studies were heterogeneous in design, purpose, population, and type of AI. This range is appropriate for a scoping review, but it limits any claim about effectiveness. Some included works were conceptual or praxis-oriented rather than empirical. They were retained because they clarify how the field frames AI, but their presence means the review should not be read as evidence that the proposed applications are empirically supported. Terminology was also inconsistent across the literature, with terms such as gifted and talented, high-ability, talent development, and twice-exceptional used in varying ways. Finally, although two reviewers screened and charted the literature independently, no formal quality appraisal of the included studies was conducted. This is consistent with the purpose of a scoping review, but it means the findings describe the available evidence rather than judge its methodological strength. In addition, several adjacent AI and gifted-education terms (e.g., intelligent tutoring systems, adaptive learning, learning analytics, deep learning, gifted identification) were used to guide screening but were not included as separate strings in the formal database search, which may have missed studies indexed only under such terminology.

## Conclusion

Artificial intelligence is becoming a visible presence in gifted and talented education, but the literature remains early, uneven, and ethically complex. The 26 studies reviewed here show two developing trajectories: instructional uses such as differentiation, writing, questioning, and creative work, and assessment-oriented uses centered on machine learning for gifted and talented and twice-exceptional identification. Across both, the field is still describing possibilities more than demonstrating outcomes.

What the literature suggests most consistently is that AI’s usefulness depends on human judgment rather than on the tools themselves. AI may ease the preparation of differentiated materials or surface patterns in complex learner profiles, but decisions about challenge, equity, and what counts as a student’s own work remain with teachers and evaluators. For a field defined by depth, creativity, and fair access, the question worth pursuing is how AI can be used responsibly to extend those aims while protecting fairness, originality, privacy, and professional judgment. Because most included studies are conceptual, praxis-oriented, or small-scale and exploratory, the patterns reported here should be read as emerging and hypothesis-generating rather than as established evidence that AI improves gifted and talented education outcomes; claims of effectiveness await stronger longitudinal, comparative, and equity-focused research.

## Data Availability

The original contributions presented in this study are included in the article/Supplementary material, further inquiries can be directed to the corresponding author.
