# Supplementary material for: Artificial intelligence in gifted and talented education: a scoping review
Source: Front Psychol. 2026 Jul 29;17:1898467. doi: 10.3389/fpsyg.2026.1898467 (PMC13463290; doi:10.3389/fpsyg.2026.1898467)
Supplement: Supplementary file 1 [file Supplementary_file_1.docx]

**Artificial Intelligence in Gifted and Talented Education: A Scoping Review**

**Supplementary Materials**

**Supplementary Table S1. Database-Specific Search Strategies and Records Retrieved**

| **Database / source** | **Date** | **Fields** | **Search string (AI AND gifted terms) / procedure** | **Limits** | **Records** |
| --- | --- | --- | --- | --- | --- |
| Web of Science (Core Collection) | 2026-05-30 | Topic (TS=) | TS=("artificial intelligence" OR "generative AI" OR ChatGPT OR "machine learning" OR "large language model" OR "neural network" OR "AI tutoring") AND TS=(gifted OR "gifted education" OR "talented education" OR "high ability" OR "high-ability" OR "twice exceptional" OR twice-exceptional OR 2e OR "talent development") | 2010-2026; English; Article, Review, Proceeding Paper, Early Access | 104 |
| Scopus | 2026-05-30 | TITLE-ABS-KEY | TITLE-ABS-KEY("artificial intelligence" OR "generative AI" OR ChatGPT OR "machine learning" OR "large language model" OR "neural network" OR "AI tutoring") AND TITLE-ABS-KEY(gifted OR "gifted education" OR "talented education" OR "high ability" OR "high-ability" OR "twice exceptional" OR twice-exceptional OR 2e OR "talent development") AND PUBYEAR > 2009 | 2010-2026; English | 113 |
| EBSCOhost (ERIC, Education Source, Academic Search Ultimate) | 2026-05-30 | Abstract (AB) | ("artificial intelligence" OR "generative AI" OR ChatGPT OR "machine learning" OR "large language model" OR "neural network" OR "AI tutoring") AND (gifted OR "gifted education" OR "talented education" OR "high ability" OR "high-ability" OR "twice exceptional" OR twice-exceptional OR 2e OR "talent development") | 01/2010-05/2026; English; peer-reviewed | 78 |
| ProQuest | 2026-05-30 | Abstract (AB) | ("artificial intelligence" OR "generative AI" OR ChatGPT OR "machine learning" OR "large language model" OR "neural network" OR "AI tutoring") AND (gifted OR "gifted education" OR "talented education" OR "high ability" OR "high-ability" OR "twice exceptional" OR twice-exceptional OR 2e OR "talent development") | 2010-05/2026; English; peer-reviewed; scholarly journals + conference papers | 102 |
| Google Scholar | 2026-05 | All fields | Targeted queries pairing AI terms with gifted/talented terms; first 50-100 results per query screened | Supplementary / verification | Screened for relevance |
| Backward & forward citation searching | 2026-05 | — | Reference lists of included studies and citations of key studies | Supplementary | As identified |
| Total database records identified |  |  |  |  | 397 |

*Note. Search strings reflect the conceptual search strategy reported in the manuscript and were adapted across databases while retaining conceptual consistency. The final search was completed in May 2026 and covered January 2010-May 2026. Google Scholar and citation searching were used for supplementary verification; the 397 records refer only to database records retrieved from Web of Science, Scopus, EBSCOhost, and ProQuest.*

**Supplementary Table S2. Full-Text Records Excluded, With Reasons (n = 41)**

| **Reason for exclusion at full-text stage** | **Records (n)** |
| --- | --- |
| AI mentioned only incidentally or no clear AI focus | 7 |
| No clear gifted/high-ability focus, including general AI-in-education or educational-technology studies | 24 |
| "Talent"/"high-achieving"/"advanced" used in a general academic sense | 5 |
| Machine-learning study on general academic prediction (not gifted) | 1 |
| Non-scholarly material | 1 |
| Not available in English | 1 |
| Insufficient information for data charting / eligibility not verifiable | 2 |
| Total excluded at full-text stage | 41 |

*Note. Of 242 records screened, 155 duplicates had been removed from 397 identified records; 175 records were excluded at title/abstract screening and 67 were assessed at full text, of which 41 were excluded for the reasons above and 26 were retained.*

**Supplementary Table S3. Expanded Data-Charting Table for All Included Studies (N = 26)**

| **#** | **Author/year** | **Country** | **Publication type** | **Evidence base** | **Population & level** | **Sample (n)** | **AI tool/model** | **Design** | **Purpose** | **Main findings / notes** | **Limitations / cautions** | **Theme** |
| --- | --- | --- | --- | --- | --- | --- | --- | --- | --- | --- | --- | --- |
| 1 | Siegle, 2023 | United States | Praxis article | Conceptual | Gifted learners (K–12) | — | ChatGPT / AI | Praxis | Differentiation support | Proposes roles for ChatGPT/AI in differentiating gifted instruction | Conceptual only | T1 |
| 2 | Siegle, 2024 | United States | Praxis article | Conceptual | Talent development (K–12) | — | AI tools | Praxis | Acceleration, depth, interests | AI to support the three legs of talent development | Conceptual only | T1 |
| 3 | Ecker et al., 2025 | United States | Theoretical framework | Conceptual | Gifted programs | — | AI tutoring | Conceptual | AI tutoring model | Four-dimensional AI tutoring framework | Conceptual framework | T1 |
| 4 | Lewis, 2025 | United States | Praxis article | Conceptual | Gifted educators | — | GenAI | Conceptual | Teacher preparation | Links AI literacy to gifted pedagogy and ethics | Conceptual / preparation-focused | T6 |
| 5 | Guilbault et al., 2025 | United States | Classroom application | Praxis-oriented | Secondary gifted students | NR | ChatGPT | Application | Personalized learning & mentoring | ChatGPT for personalization and virtual mentoring | NR sample; practice-based | T1 |
| 6 | Siegle, 2025 | United States | Instructional strategy | Conceptual | Gifted students | — | Prompt engineering | Conceptual | Improve questioning | Prompt engineering/chaining to strengthen questioning | Conceptual strategy | T3 |
| 7 | Tamim, 2025 | Lebanon | Mixed methods | Empirical | Gifted & average-IQ students | NR | AI / ChatGPT | Mixed methods | Compare AI use | Gifted students use AI for learning and self-exploration | NR sample; context-specific | T1, T3 |
| 8 | Bedir et al., 2025 | Türkiye | Qualitative | Empirical | Gifted students; science-and-art centers | NR | AI | Qualitative drawings | Explore perceptions | Maps gifted students’ AI perceptions through drawings | NR sample; perception-based | T3 |
| 9 | Mohamed et al., 2026 | Saudi Arabia | Quantitative correlational | Empirical | Gifted EFL university students | NR | GenAI | Correlational | Motivation & anxiety | Gender and AI anxiety moderate/mediate motivation links | Correlational; NR sample | T1 |
| 10 | Hasanbaşoğlu & Baloğlu, 2026 | Türkiye | Qualitative | Empirical | Gifted college students | NR | ChatGPT | Qualitative | Academic writing | Use for planning, drafting, revision; integrity concerns | NR sample; self-reported use | T2 |
| 11 | Li et al., 2025 | Taiwan | Comparative quantitative | Empirical | Gifted & non-gifted students | NR | AI | Comparative | Scientific inquiry | Gifted students show higher AI literacy and safer-use confidence | NR sample; comparative context | T3 |
| 12 | Alkan, 2026 | Türkiye | Exploratory pre–post | Empirical | Gifted students | NR | AI / ML gamified | Pre–post | Cognitive load | Examines gamified AI effects on cognitive load | Exploratory; NR sample | T1, T3 |
| 13 | Almumen & Jouhar, 2025 | Kuwait | Single-case design | Empirical | Gifted students; incl. 1 2e | 5 | ChatGPT 3.5 | Single-case | Persuasive writing | Brainstorming improved persuasive writing across participants | Small sample; single-case | T2 |
| 14 | Doğan & Kahraman, 2025 | Türkiye | Case study | Empirical | Gifted fourth graders | NR | ChatGPT / AI-generated STEM | Pre–post + interviews | Scientific creativity | AI-generated STEM activities improved scientific creativity | NR sample; short-term | T3 |
| 15 | Chernenko & Lysenko, 2025 | Ukraine | Conference paper | Empirical | Gifted youth | NR | AI projects | Empirical | Cognitive competence | AI project work linked to creative and analytical skills | Conference paper; NR sample | T3 |
| 16 | Ronksley-Pavia et al., 2025 | Australia | Exploratory empirical | Empirical | 2e / multi-exceptional learners | NR | GenAI / LLMs | Exploratory | Personalized learning | Synthetic learner profiles support planning for 2e learners | Exploratory; synthetic profiles | T5, T1 |
| 17 | Mahmoudi-Dehaki & Nasr-Esfahani, 2025 | Iran | Mixed-methods intervention | Empirical | 2e bilingual students | 12 | Human–AI tutoring | Quasi-exp + qual | Writing support | Hybrid tutoring improved writing-related outcomes | Small sample; specific 2e group | T5, T2 |
| 18 | Atmaca & Baloğlu, 2025 | Türkiye | Assessment development | Empirical | TD, gifted, 2e, SLD students | NR | CART / ML | Instrument development | 2e identification | Developed ML-based battery for twice-exceptionality | NR sample; validation-dependent | T4, T5 |
| 19 | Hodges & Mohan, 2019 | United States | Methodological demonstration | Empirical | Gifted identification | NR | Neural networks | Demonstration | Classification demonstration | Demonstrates neural-network classification; cautions use | Demonstration; not implementation | T4 |
| 20 | Lee et al., 2025 | South Korea | ML analysis | Empirical | Elementary students | 1157 | GMM / SVM / MLP | ML analysis | Improve identification | ML can cluster gifted students; reveals rating bias | Dataset-specific; fairness concerns | T4 |
| 21 | Önal et al., 2026 | Türkiye | Mixed methods | Empirical | BILSEM teachers | NR | AI | Mixed methods | AI self-efficacy | High AI self-efficacy; ethical caution; need for PD | NR sample; self-report | T6 |
| 22 | Al-Bawaleez & Turkey, 2025 | Jordan | Qualitative | Empirical | Gifted education institutes | NR | AI | Qualitative | Institutional practice | AI seen as useful for administrative efficiency | NR sample; context-specific | T6 |
| 23 | Akdeniz et al., 2025 | Türkiye/USA | Empirical evaluation | Empirical | Gifted education context | NR | ChatGPT / Bard–Gemini | Evaluation | Generate Bloom questions | AI stronger at lower Bloom levels; humans stronger at higher levels | Tool-specific; evaluation-focused | T3, T6 |
| 24 | García-López et al., 2025 | Spain | Systematic review | Review | Gifted EFL / EL classrooms | — | AI | Systematic review | Review language-learning uses | Synthesizes AI uses in gifted EFL/EL classrooms | Secondary evidence | T2 |
| 25 | Rubenstein et al., 2025 | United States | Action research | Empirical | Grades 3–4; Project Brilliance | NR | ChatGPT | Action research | Personalized playscripts | Teachers generate interest-based playscripts | Action research; NR sample | T2, T1 |
| 26 | Thompson et al., 2025 | United States | Quasi-experimental | Empirical | Advanced virtual science classroom | NR | GPT-4o / LLM | Quasi-experimental | Co-tutoring impact | No significant outcome differences; needed clearer structure | NR sample; short-term outcome | T1 |

Note. Evidence base classifies each study as empirical, conceptual/praxis, or review. “NR” = not reported or not applicable; “—” indicates conceptual or review work without a participant sample. “Limitations / cautions” summarizes reporting or interpretive cautions relevant to evidence mapping and should not be read as a formal quality appraisal. Theme codes: T1 = personalized/differentiated learning; T2 = writing/language/literacy; T3 = creativity, questioning, higher-order thinking; T4 = identification, assessment, decision support; T5 = twice-exceptional learners; T6 = teacher readiness, institutions, ethics.

**Supplementary Table S4. PRISMA-ScR Checklist**

| **#** | **PRISMA-ScR item** | **Reported** | **Location** |
| --- | --- | --- | --- |
| 1 | Title — identified as a scoping review | Yes | Title |
| 2 | Structured summary (abstract) | Yes | Abstract |
| 3 | Rationale | Yes | Introduction |
| 4 | Objectives / research questions | Yes | Introduction (RQ1–RQ5) |
| 5 | Protocol and registration | No registered protocol | Methods / Limitations |
| 6 | Eligibility criteria | Yes | Methods — Eligibility Criteria |
| 7 | Information sources | Yes | Methods — Information Search Strategy |
| 8 | Search (full strategy) | Yes | Methods + Supplementary Table S1 |
| 9 | Selection of sources of evidence | Yes | Methods — Screening and Study Selection |
| 10 | Data charting process | Yes | Methods — Data Charting and Synthesis |
| 11 | Data items | Yes | Methods + Supplementary Table S3 |
| 12 | Critical appraisal (optional for ScR) | Not conducted | Methods/Limitations (no formal appraisal, per ScR guidance) |
| 13 | Synthesis of results | Yes | Methods — Data Charting and Synthesis; Results |
| 14 | Selection of sources of evidence (flow) | Yes | Results — Figure 1 (PRISMA) + Supplementary Table S2 |
| 15 | Characteristics of sources of evidence | Yes | Results — Table 1, Figure 2, Supplementary Table S3 |
| 16 | Critical appraisal within sources | Not conducted | Not conducted (ScR) |
| 17 | Results of individual sources of evidence | Yes | Results — Table 1 + Supplementary Table S3 |
| 18 | Synthesis of results | Yes | Results — Thematic Findings; Table 2; Figures 3–4 |
| 19 | Summary of evidence | Yes | Discussion |
| 20 | Limitations | Yes | Discussion — Limitations |
| 21 | Conclusions | Yes | Conclusion |
| 22 | Funding | Yes | Declarations — Funding |

**Supplementary Table S5. Thematic Matrix: Studies Contributing to Each Theme and Evidence Profile**

| **Theme** | **Contributing studies (author, year)** | **Evidence profile** |
| --- | --- | --- |
| T1 Personalized & differentiated learning | Siegle (2023); Siegle (2024); Ecker et al. (2025); Guilbault et al. (2025); Tamim (2025); Mohamed et al. (2026); Alkan (2026); Ronksley-Pavia et al. (2025); Rubenstein et al. (2025); Thompson et al. (2025) | Mixed: conceptual/praxis plus small-scale empirical |
| T2 Writing, language & literacy | Hasanbaşoğlu & Baloğlu (2026); Almumen & Jouhar (2025); Mahmoudi-Dehaki & Nasr-Esfahani (2025); García-López et al. (2025); Rubenstein et al. (2025) | Empirical (small-scale) plus one systematic review |
| T3 Creativity, questioning & higher-order thinking | Siegle (2025); Tamim (2025); Bedir et al. (2025); Li et al. (2025); Doğan & Kahraman (2025); Chernenko & Lysenko (2025); Akdeniz et al. (2025) | Mostly empirical (small-scale/exploratory) |
| T4 Identification, assessment & decision support | Atmaca & Baloğlu (2025); Hodges & Mohan (2019); Lee et al. (2025) | Empirical (machine learning) |
| T5 Twice-exceptional & multi-exceptional learners | Ronksley-Pavia et al. (2025); Mahmoudi-Dehaki & Nasr-Esfahani (2025); Atmaca & Baloğlu (2025) | Empirical (exploratory/small-sample) |
| T6 Teacher readiness, institutions & ethics | Lewis (2025); Önal et al. (2026); Al-Bawaleez & Turkey (2025); Akdeniz et al. (2025) | Mixed: conceptual plus empirical survey/qualitative |
